# The functionalized amino acid (*S*)-Lacosamide subverts CRMP2-mediated tubulin polymerization to prevent constitutive and activity-dependent increase in neurite outgrowth

**DOI:** 10.3389/fncel.2014.00196

**Published:** 2014-07-24

**Authors:** Sarah M. Wilson, Aubin Moutal, Ohannes K. Melemedjian, Yuying Wang, Weina Ju, Liberty François-Moutal, May Khanna, Rajesh Khanna

**Affiliations:** ^1^Paul and Carole Stark Neurosciences Research Institute, Indiana University School of MedicineIndianapolis, IN, USA; ^2^Department of Pharmacology, College of Medicine, University of ArizonaTucson, AZ, USA; ^3^Department of Neurology, The First Hospital of Jilin University, and Jilin UniversityJilin, China; ^4^Neuroscience Graduate Interdisciplinary Program, College of Medicine, University of ArizonaTucson, AZ, USA

**Keywords:** CRMP2, activity-dependent, neurite outgrowth, (*S*)-Lacosamide, GSK3β, Cdk5

## Abstract

Activity-dependent neurite outgrowth is a highly complex, regulated process with important implications for neuronal circuit remodeling in development as well as in seizure-induced sprouting in epilepsy. Recent work has linked outgrowth to collapsin response mediator protein 2 (CRMP2), an intracellular phosphoprotein originally identified as axon guidance and growth cone collapse protein. The neurite outgrowth promoting function of CRMP2 is regulated by its phosphorylation state. In this study, depolarization (potassium chloride)-driven activity increased the level of active CRMP2 by decreasing its phosphorylation by GSK3β via a reduction in priming by Cdk5. To determine the contribution of CRMP2 in activity-driven neurite outgrowth, we screened a limited set of compounds for their ability to reduce neurite outgrowth but not modify voltage-gated sodium channel (VGSC) biophysical properties. This led to the identification of (*S)-lacosamide* ((*S*)-LCM), a stereoisomer of the clinically used antiepileptic drug (*R)-LCM* (Vimpat®), as a novel tool for preferentially targeting CRMP2-mediated neurite outgrowth. Whereas (*S*)-LCM was ineffective in targeting VGSCs, the presumptive pharmacological targets of (*R*)-LCM, (*S*)-LCM was more efficient than (*R*)-LCM in subverting neurite outgrowth. Biomolecular interaction analyses revealed that (*S*)-LCM bound to wildtype CRMP2 with low micromolar affinity, similar to (*R*)-LCM. Through the use of this novel tool, the activity-dependent increase in neurite outgrowth observed following depolarization was characterized to be reliant on CRMP2 function. Knockdown of CRMP2 by siRNA in cortical neurons resulted in reduced CRMP2-dependent neurite outgrowth; incubation with (*S*)-LCM phenocopied this effect. Other CRMP2-mediated processes were unaffected. (*S*)-LCM subverted neurite outgrowth not by affecting the canonical CRMP2-tubulin association but rather by impairing the ability of CRMP2 to promote tubulin polymerization, events that are perfunctory for neurite outgrowth. Taken together, these results suggest that changes in the phosphorylation state of CRMP2 are a major contributing factor in activity-dependent regulation of neurite outgrowth.

## Introduction

Neurite outgrowth is a highly regulated, progressive process, responding to a myriad of intrinsic as well as external cues, such as network activity. Activity-dependent outgrowth has long been attributed to focal changes in intracellular calcium concentration (Cohan and Kater, 1986; Connor, 1986; Fields et al., 1990; Schilling et al., 1991). The growth promoting aspect of activity is constrained within a small range of calcium concentrations, outside of which retraction and growth cone collapse can occur, suggesting the process is tightly regulated (Cohan and Kater, 1986; Kater et al., 1988; van Pelt et al., 1996). Such regulation allows for a reciprocal relationship between outgrowth and network connectivity (Van Ooyen et al., 1995). A better understanding of activity-dependent changes in synaptic organization may provide essential insight into pathological processes involving abnormally high levels of neuronal activity, such as epilepsy.

Mechanistic studies have suggested that the rise in intracellular calcium necessary to promote growth may be attributed to calcium influx through N-methyl-D-aspartate receptors, L-type voltage-gated calcium channels, or both (Kocsis et al., 1994; Wayman et al., 2006). Regardless of the initial route of calcium entry, preventing its secondary mobilization from intracellular stores abolishes the growth promoting effects of depolarization-induced activity (Kocsis et al., 1994). Other than strict calcium dependence, the specific mechanisms underlying activity-dependent outgrowth are relatively unknown. Second messenger systems, especially those responsive to changes in intracellular calcium such as cAMP, have been suggested to play a role (Mattson et al., 1988). Additionally, inhibitors of transcription have also been used to suggest that transcription—most likely of growth promoting genes—may be an important step linking activity to cytoskeletal dynamics (Solem et al., 1995). Further studies also suggest the importance of kinase cascades (Solem et al., 1995; Wayman et al., 2006). Recently, activity-induced outgrowth in cerebellar granule cells was attributed to changes in the kinase GSK3β and its substrate, collapsin response mediator protein 2 (CRMP2) (Tan et al., 2013). CRMP2 is an intracellular phosphoprotein originally identified for coordinating axon guidance and growth cone collapse (Goshima et al., 1995). More recently, numerous other functions for CRMP2 have been identified, implicating CRMP2 in a variety of neuropathologies (for review see Hensley et al., 2011; Khanna et al., 2012).

Of particular importance to our investigations is the ability of CRMP2 to mediate neurite outgrowth via transport and stabilization of tubulin dimers (Fukata et al., 2002) as well as enhancement of tubulin's intrinsic GTPase activity (Chae et al., 2009). In regards to its outgrowth-promoting function, the activity of CRMP2 is regulated by its phosphorylation state. In the unphosphorylated state CRMP2 is considered active and thereby growth-promoting, however, upon phosphorylation by glycogen synthase kinase 3β (GSK3β), cyclin dependent kinase 5 (Cdk5), Rho kinase (RhoK), Ca^2+^ /calmodulin-dependent protein kinase II (CaMKII), or Fyn kinases, CRMP2 is rendered inactive (Arimura et al., 2000, 2005; Brown et al., 2004; Cole et al., 2004, 2006; Uchida et al., 2005, 2009; Yoshimura et al., 2005; Hou et al., 2009). Along those same lines, dephosphorylation by the protein phosphatases PP2A and PP1 promotes neurite outgrowth (Zhu et al., 2010; Astle et al., 2011). Elegant studies by the Ohshima and Goshima groups suggest that changes in the phosphorylation state of CRMP2 may allow for dynamic regulation of outgrowth and branching patterns, as phosphorylation by Cdk5 is necessary for proper bifurcation of CA1 apical dendrites as well as organization of dendritic fields (Yamashita et al., 2012; Niisato et al., 2013). In fact, the ability of CRMP2 to coordinate axon guidance/growth cone collapse by Sema3A relies on phosphorylation (Arimura et al., 2005; Uchida et al., 2005, 2009). However, very little is known about the regulation of CRMP2 phosphorylation following neuronal activity. As post-translational modifications serve to regulate numerous processes throughout the nervous system, including those that are activity-dependent, here we tested the hypothesis that changes in CRMP2 phosphorylation may account for the changes in neurite outgrowth observed following neuronal activity.

In this study we demonstrate that neuronal activity induced by KCl depolarization in cortical neurons led to decreased GSK3β phosphorylation (residues T509/T514) of CRMP2, which was attributed to reduced priming by Cdk5 (residue S522 of CRMP2). The loss of phosphorylation directly translated into an increase in the amount of CRMP2/tubulin binding, concomitant with an increase in neurite outgrowth. Through the use of a novel small molecule identified herein, we were able to demonstrate an inhibition in CRMP2-facilitated tubulin polymerization without a change in CRMP2-tubulin binding. Consequently, it was determined that activity-driven neurite outgrowth was prevented through inhibition of CRMP2-mediated neurite outgrowth.

## Materials and methods

### Materials

All reagents were purchased from Sigma (St. Louis, MO, USA) unless otherwise indicated. *(S)*-LCM, a functionalized amino acid, and some of its derivatives were provided by the laboratory of Dr. Harold Kohn, University of North Caroline, Chapel Hill. A 100 mM solution was made up in dimethylsulfoxide (DMSO) and stored in small aliquots at −20°C. Microscale thermophoresis (MST) labeling solutions and binding buffers were purchased from Nanotemper (München, Germany).

### Primary cortical neuron culture

Primary cortical neurons were prepared from embryonic day 18–19 Sprague–Dawley rats as described (Brittain et al., 2009, 2011a; Wang et al., 2010a) with some modifications. Briefly, cortices were dissected and cell suspensions were plated onto poly-D Lysine-coated glass coverslips. 5-fluor-2′-deoxyuridine (1.5 μg/mL) was added 48 h after plating to reduce the number of non-neuronal cells. The cultures contain ~95% neurons (both excitatory and inhibitory) with the remaining 5% comprised mostly of astrocytes and the occasional microglia. All procedures were in compliance with Institutional Animal Care and Use Committees of the Indiana University School of Medicine and the College of Medicine at the University of Arizona as well as with ARRIVE guidelines.

### Immunoblot analysis

Lysates were made from cultured cortical neurons at 6 DIV in RIPA buffer containing protease and phosphatase inhibitor cocktails as previously described (Brittain et al., 2009). Samples were boiled in Laemmli sample buffer and separated by electrophoresis on SDS-polyacrylamide gels. Proteins were transferred to polyvinylidene difluoride membranes and blocked at room temperature for 1 h and incubated in primary antibodies CRMP2 (Cat# C2993, Sigma, St. Louis, MO), CRMP2 pThr509/Thr514 (Cat# PB-043, Kinasource, Dundee, Scotland, UK), CRMP2 pSer522 (Cat# CP2191, ECM Biosciences, Versailles, KY), GSK3β pSer9 (Cat# 5558, Cell Signaling, Danvers, MA), Cdk5 (Cat# 2506, Cell Signaling, Danvers, MA), p35 (Cat# 2680, Cell Signaling, Danvers, MA), GSK3β (Cat# 9832, Cell Signaling, Danvers, MA), or Tubulin (Cat# G712A, Promega, Madison, WI) overnight at 4°C. Following incubation in horseradish peroxidase conjugated secondary antibodies, blots were probed with enhanced chemiluminescence Western blotting substrate (Thermo Scientific, Waltham, MA) before exposure to photographic film. Films were scanned, digitized, and quantified using Un-Scan-It gel version 6.1 scanning software (Silk Scientific Inc, Orem, UT).

### Neurite outgrowth imaging and analysis

Primary cortical neurons were transfected via lipofectamine 2000 (Invitrogen) with enhanced green fluorescent protein (EGFP) at 4 DIV and incubated 24 h in 300 μ M (*R*)-LCM, LCM derivatives, or vehicle before imaging with ImageXpress Micro (Molecular Devices, Sunyvale, CA). The overexpression of EGFP allowed for visualization of a small percentage of neurons while maintaining optimal cell densities required for survival. To compare the effects of (*S*)-LCM and CRMP2siRNA primary cortical neurons were transfected via lipofectamine 2000 with EGFP, control siRNA + EGFP, or CRMP2-siRNA + EGFP at 4 DIV and incubated with 200 μM (*S*)-LCM for 24 h before imaging with ImageXpress Micro. Analysis of neurite outgrowth was completed using a neurite outgrowth analysis protocol with the MetaXpress software (Molecular Devices, Sunnyvale, CA). Non-neuronal cells were easily identifiable and excluded based on morphology. Neuronal cell soma and processes are detected by defining separate size and fluorescence intensity threshold parameters. Maximum width and minimum area parameters for determining somas were set to 50 and 300 μm^2^, respectively. For identifying processes, maximum width and minimum length parameters were set to 8 and 3 μm, respectively. Cells were excluded if they were determined not to be neurons based on morphology, if processes extended beyond the image field, or if no processes were longer than 50 μm. The following parameters are recorded and summarized into a final “total outgrowth” parameter: number of processes, number of branches, mean process length, and maximum process length.

### Purification of recombinant CRMP2 and CRMP2_5ALA_ proteins

A CRMP2-GST construct containing 5 amino acids in predicted LCM-binding regions of CRMP2 mutated to alanine (CRMP2_5ALA_GST; G360, S363, K418, I420, and P443) was generated by subcloning the mutation containing portion of CRMP2_5ALA_-EGFP into wild-type CRMP2-GST (using restriction enzymes *RsrII* and *BglII*). Both wild-type and mutant recombinant proteins were purified exactly as described (Brittain et al., 2009; Wang et al., 2010b).

### Microscale thermophoresis (MST) binding analysis

MST, the directed movement of molecules in optically generated microscopic temperature gradients, permits an immobilization-free fluorescence methodology for the analysis of biomolecular interaction (Wienken et al., 2010; van den Bogaart et al., 2012). The thermophoretic movement is determined by the entropy of the hydration shell around the labeled molecule. The solvation entropy and the hydration shell of the macromolecules provide the driving force for the flow, and any change of the hydration shell of biomolecules due to changes in their primary, secondary, tertiary and/or quaternary structure affects their thermophoretic mobility and can be used to determine binding affinities and enzymatic activities with high accuracy and sensitivity. A microscopic temperature gradient is generated by an infrared laser. In a typical MST-experiment the concentration of the labeled molecule is kept constant while the concentration of the unlabeled interaction partner is varied. NT647-labeled CRMP2 or CRMP2_5ALA_ (500 nM final) was incubated for 10 min at room temperature in the dark with increasing concentrations of *(S)*-LCM. Thermophoresis analysis was performed on a NanoTemper Monolith NT.115 instrument (25% LED; 25% IR-laser power). The MST curves were fitted with a Hill method using Origin 8.5 software to obtain apparent Kd values for binding interactions.

### ELISA-based CRMP2-tubulin binding assay

The 96-well plates (Nunc Maxisorp, Thermo Scientific) were coated with tubulin (200 ng/well, Cytoskeleton Inc.) and incubated at room temperature overnight. The following day the plates were washed and blocked with 3% BSA to minimize non-specific adsorptive binding to the plates. Escalating concentrations of CRMP2 were added to the plates. As a negative control, some wells received escalating concentrations of CRMP2 which was denatured by heating at 95°C for 5 min. The plates were incubated at room temperature with shaking for 2 h. The plates were then washed with PBS containing 0.5% Tween-20 to eliminate unbound CRMP2. The bound CRMP2 was detected by CRMP2 primary antibody (150 ng/ml, Sigma) followed by HRP-conjugated secondary antibody (GE Healthcare). Tetramethylbenzidine (R&D Systems) was used as the colorimetric substrate. The optical density of each well was determined immediately, using a microplate reader (Multiskan Ascent, Thermo) set to 450 nm with a correction wavelength of 570 nm. Data was analyzed by non-linear regression analysis using GraphPad Prism5 (Graph Pad, San Diego, CA).

To determine the effect of (*S*)-LCM on CRMP2-tubulin binding, 96-well plates were coated with tubulin and incubated overnight. The following day the plates were washed and blocked with 3% BSA to minimize non-specific adsorptive binding to the plate. After the wash all the wells received 1 μM of CRMP2 and none or escalating concentrations of (*S*)-LCM. The plates were then incubated at room temperature with shaking for 2 h. This was followed with washes to eliminate the unbound CRMP2. CRMP2 bound to the plates was detected using the method described above.

A cell lysate based ELISA was performed as described above, with some modifications. In tubulin-coated 96-well plates, lysates from cortical cells treated with vehicle, KCl, (*S*)-LCM, or KCl + (*S*)-LCM were added. Next, the plates were incubated at room temperature with shaking for 2 h. The plates were then washed to eliminate the unbound proteins. The bound CRMP2 was detected as described above. The optical density of each well was determined immediately, using a microplate reader set to 450 nm with a correction wavelength of 570 nm. The optical densities were normalized to the amount of protein in each sample. Data was analyzed as described earlier.

### Turbidimetric assay for tubulin polymerization

Polymerization of tubulin was performed as previously described (Chae et al., 2009; Wilson et al., 2012a) with modifications. Polymerization was performed in 0.1 M G-PEM buffer (1 mM GTP, 80 mM PIPES, 1 mM EGTA, 1 mM MgCl_2_, pH 7.0), 1 mM Na-GTP (Sigma), and 2 mg/ml tubulin (Cytoskeleton, Inc). CRMP2 proteins (0.2 μM) as well as 3 μM of (*R*)-LCM, (*S)-LCM* or 0.01% DMSO were added to the samples and pipetted onto a 96-well plate at 4°C. Following a 30 min incubation on ice, turbidity changes were assessed at 412 nm using a Synergy™ 2 Multi-Detection Microplate Reader (BioTek Instruments, Inc., San Diego, CA) which had previously been pre-warmed to 37°C. Absorbances were measured over time and compared to background samples which contained only buffer + GTP.

### Whole-cell patch-clamp recordings

Whole-cell voltage recordings were performed at room temperature on primary cultured cortical neurons using an EPC 10 Amplifier (HEKA Electronics, Germany). Electrodes were pulled from thin-walled borosilicate glass capillaries (Warner Instruments, Hamden, CT) with a P-97 electrode puller (Sutter Instrument, Novato, CA) such that the final electrode resistances were 2–3 MΩ when filled with internal solutions. The internal solution for recording Na^+^ currents contained (in mM): 110 CsCl, 5 MgSO_4_, 10 EGTA, 4 ATP Na_2_, and 25 HEPES (pH 7.2, 290-310 mOsmo/L). For recording Na^+^ currents, the external solution contained (in mM): 100 NaCl, 10 tetraethylammonium chloride (TEA-Cl), 1 CaCl_2_, 1 CdCl_2_, 1 MgCl_2_, 10 D-glucose, 4 4-AP, 0.1 NiCl_2_, 10 HEPES (pH 7.3, 310-315 mOsm/L). Whole-cell capacitance and series resistance were compensated with the amplifier (70–80%). Cells were considered only when the seal resistance was more than 1 GΩ and the series resistance was less than 10 MΩ. Linear leak currents were digitally subtracted by P/4. Neurons were held at −100 mV, conditioned to potentials ranging from −10 to +20 mV (in +10 mV increments) for 5 s, and then fast-inactivated channels were allowed to recover for 150 ms at a hyperpolarized pulse to −120 mV, and the fraction of channels available was tested by a single depolarizing pulse, to 0 mV, for 15 ms.

### Knockdown of CRMP2 expression by siRNA

Validated short interfering RNAs (siRNAs) against the rat CRMP2 (5′- ACTCCTTCCTCGTGTACAT-3′) sequence (Brittain et al., 2009) and controls (scrambled sequence with approximately the same GC percentage but no sequence homology) were used for CRMP2 knockdown (Invitrogen) in cortical neurons as described (Brittain et al., 2009; Chi et al., 2009; Wilson et al., 2012a). Cells were incubated for 2 days with vector- or scramble siRNA (250 nM) and extent of knockdown was assessed via immunoblot analysis. As previously reported (Chi et al., 2009; Brustovetsky et al., 2014), we observed ~90% knockdown of CRMP2 compared to scramble siRNA (data not shown).

### Activity-dependent neurite outgrowth imaging

For analysis of activity-dependent outgrowth, neurons were incubated in vehicle (<0.01% DMSO), 25 mM KCl, 200 μM *(S)*-LCM, or 200 μM (*S*)-LCM + 25 mM KCl for 96 h beginning at 2 DIV. Media was changed at 4 DIV, maintaining KCl levels at 25 mM, at which time neurons were also transfected with EGFP (4 DIV). EGFP-expressing cells were then imaged at 6 DIV. The use of 25 mM KCl is consistent with previous work reporting the involvement of CRMP2 in activity-dependent outgrowth (Tan et al., 2013). Analysis of neurite outgrowth was completed using a neurite outgrowth analysis protocol with the MetaXpress software (Molecular Devices, Sunnyvale, CA).

### Data analysis

All data points are shown as mean ± s.e.m. Statistical differences between control and experimental conditions were determined by using ANOVA with a Dunnett's or Tukey's *post-hoc* test or a Student's *t*-test when comparing only two conditions. Values of *p* < 0.05 were judged to be statistically significant.

## Results

### Moderate activity reduces CRMP2 phosphorylation by GSK3β without affecting kinase activity

**Figure 1:**
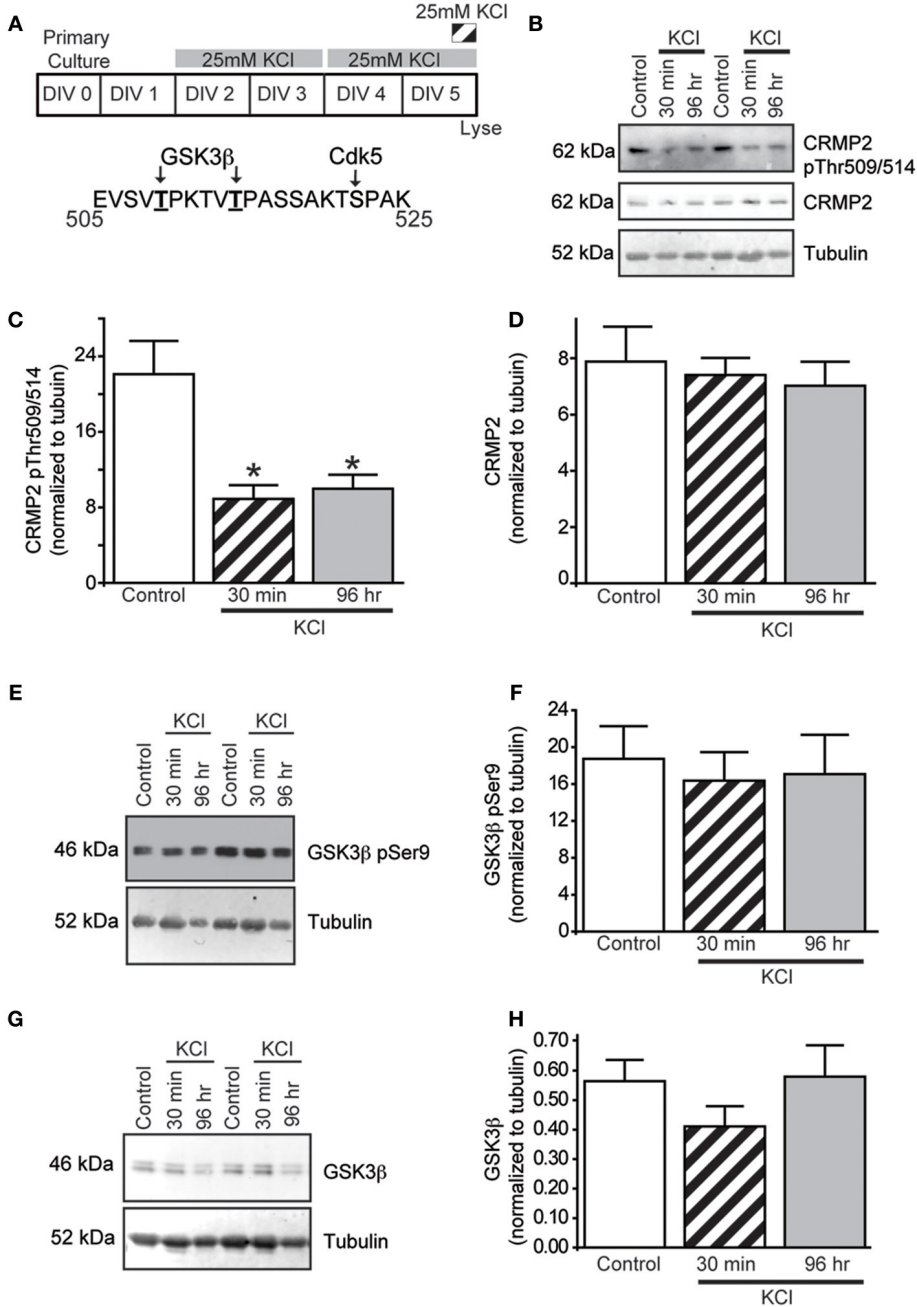
**KCl-induced activity decreases GSK3 β phosphorylation of CRMP2 without changing GSK3 β activity or expression. (A)** Top: Timeline of experimental procedures. Bottom: Schematic of GSK3β and Cdk5 phosphorylation sites within the rat CRMP2 sequence. Numbers represent amino acid residues within the CRMP2 sequence. Representative immunoblots of GSK3β-phosphorylated CRMP2 (CRMP2 pThr509/pThr514), total CRMP2, and β III-tubulin **(B)**, inactivated Ser9-phosphorylated GSK3β and β III-tubulin **(E)**, and total GSK3β and β III-tubulin **(G)** from naïve cortical neurons compared to those exposed to KCl for 30 min or 96 h. Summary of the relative levels of the indicated proteins **(C,D,F,H)**. Expression of GSK3β-phosphorylated CRMP2 (CRMP2 pThr509/pThr514) is decreased following 30 min or 96 h exposure to KCl **(C)** whereas total CRMP2 expression was not affected by KCl exposures **(D)** (^*^p < 0.05 compared to control; One-Way ANOVA, Tukey's *post-hoc* analysis) (*n* = 4). KCl treatment did not alter phosphorylation of GSK3β **(F)** nor total GSK3β expression **(H)** did not change following 30 min or 96 h KCl treatment (One-Way ANOVA, Tukey's *post-hoc* analysis) (*n* = 5).

CRMP2 has recently been suggested as a potential mediator of activity-dependent neurite outgrowth in cerebellar granule neurons (Tan et al., 2013). Unlike other central neurons, cerebellar granule cells require slightly depolarizing conditions for survival *in vitro*. Therefore, it is difficult to generalize this finding to other neuronal populations within the central nervous system. As such, it is not known if CRMP2 is involved in outgrowth induced by depolarization in neurons where it is not necessary for survival. As the ability of CRMP2 to enhance neurite outgrowth is highly dependent upon its phosphorylation state (for review see Khanna et al., 2012), Western blot analysis was used to determine the level of GSK3β-phosphorylated CRMP2 following acute (30 min) and chronic exposure to KCl (96 h) treatment (Figure 1A). The 96 h KCl treatment was chosen as it reproducibly increased neurite outgrowth. Additionally, as it was unknown how long any biochemical changes, such as phosphorylation, would be sustained, a more acute (30 min) treatment was also used. Treatment with 25 mM KCl reduced the level of GSK3β-phosphorylated CRMP2 by ~60.8% (acute) and ~54.8% (chronic) compared to control (*p* < 0.05), while total CRMP2 expression did not change (Figures 1B–D). Therefore, exposure to KCl leads to increased levels of active, unphosphorylated CRMP2.

Similar to CRMP2, GSK3β activity is regulated by changes in phosphorylation state, whereby phosphorylation at GSK3β Ser9 is sufficient to inhibit its kinase function (Cross et al., 1995). To determine if the decrease in CRMP2 phosphorylation induced by KCl is due to decreased levels of GSK3β activity, the amount of Ser9-phosphorylated GSK3β was measured following KCl exposure. Interestingly, neither Ser9 phosphorylation nor total expression of GSK3β was affected by KCl exposure (Figures 1E–H), suggesting that the decrease in GSK3β-phosphorylated CRMP2 is not attributed to a change in GSK3β expression or activity.

### Moderate activity reduces Cdk5 priming of CRMP2

**Figure 2:**
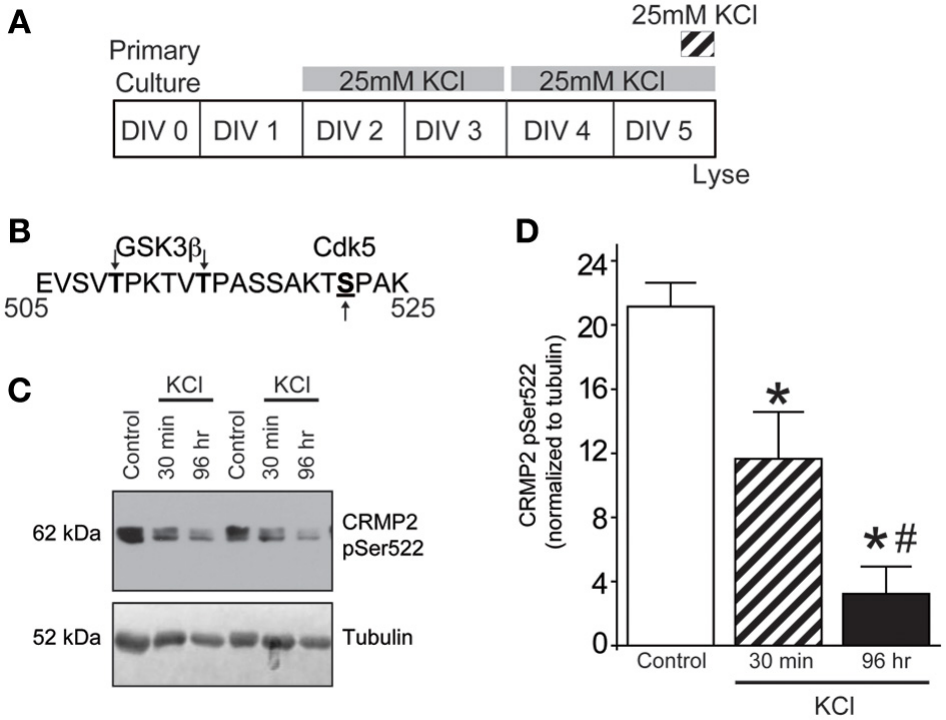
**KCl-induced activity decreases Cdk5-phosphorylated CRMP2. (A)** Timeline of experimental procedures. **(B)** Schematic of GSK3β and Cdk5 phosphorylation sites within the rat CRMP2 sequence. Numbers represent amino acid residues within the CRMP2 sequence. **(C)** Representative immunoblots of Cdk5-phosphorylated CRMP2 (CRMP2 pSer522) and β III-tubulin from naïve cortical neurons compared to those exposed to KCl for 30 min or 96 h. **(D)** KCl exposure lead to a time-dependent decrease in the level of Cdk5-phosphorylated CRMP2 (CRMP2 pSer522) (^*^p < 0.05 compared to control and ^#^*p* < 0.05 compared to 30 min KCl treatment; One-Way ANOVA, Tukey's *post-hoc* analysis; *n* = 5).

In the case of CRMP2, substrate recognition by GSK3β first requires phosphorylation by Cdk5 at a downstream site (Ser522), which “primes” the protein for subsequent GSK3β phosphorylation (Cole et al., 2006) (see Figure 2B). Therefore, Western blot analysis of Cdk5-phosphorylated CRMP2 was used to determine if the KCl-induced decrease in GSKβ phosphorylation is due to a reduction in Cdk5 priming. Both acute and chronic exposure to KCl (Figure 2A) decreased the level of Cdk5-phosphorylated CRMP2 in a time dependent manner by ~44.8% (acute) and ~84.9% (chronic) compared to control (*p* < 0.05) (Figures 2C,D), suggesting that the activity-dependent decrease in GSK3β-phosphorylated CRMP2 can be attributed to decreased levels of Cdk5-primed CRMP2.

The loss of Cdk5-phopshorylated CRMP2 cannot be attributed to changes in kinase expression as levels of Cdk5 remained consistent following KCl exposure (0.0159 ± 0.0012) compared to control (0.0156 ± 0.0004) (*p* > 0.05) (Supplemental Figure 1). Aside from expression level, Cdk5 activity is primarily determined by the level of its cofactor p35 (Lee et al., 1996; Zhu et al., 2005). However, Western blot analyses reveals that expression of p35 was also not altered following KCl exposure (0.0113 ± 0.0014) compared to control (0.0110 ± 0.0009) (*p* > 0.05), suggesting the loss of Cdk5-phosphorylated CRMP2 is independent of the expression of Cdk5 or its cofactor.

### Identification of an (R)-LCM derivative for the study of CRMP2-mediated neurite outgrowth

The aforementioned data suggest that chronic depolarization via KCl leads to a prolonged loss of both GSK3β- and Cdk5-phosphorylated CRMP2. As phosphorylation at these sites is canonically known to inactivate the outgrowth-promoting function of CRMP2 (Brown et al., 2004; Cole et al., 2004, 2006; Uchida et al., 2005; Yoshimura et al., 2005), the KCl-induced loss of phosphorylation should translate to an overall increase in CRMP2 activity. However, to determine how the increase in the proportion of active CRMP2 relates to KCl-facilitated neurite outgrowth, it is necessary to preferentially target CRMP2 function. Previously, we demonstrated that the novel anti-epileptic drug (2R)-2-(acetylamino)-N-benzyl-3-methoxypropanamide [(*R*)-LCM, tradename Vimpat®] could directly impair the ability of CRMP2 to enhance neurite outgrowth (Wilson et al., 2012a). (*R*)-LCM purportedly targets steady-state gating kinetics by selectively enhancing sodium channel slow inactivation (Zhu et al., 2005). However, its additional actions on the voltage gated sodium channel (VGSC) render it unsuitable for use as a tool in investigation of CRMP2 functions (Errington et al., 2008; Sheets et al., 2008). Therefore, derivatives of (*R*)-LCM that were unable to impact VGSC slow-inactivation were screened for their ability to inhibit neurite outgrowth (Table 1). Neurite outgrowth was measured from EGFP-transfected primary cultured cortical neurons using the ImageXpress Micro and MetaXpress software systems (Molecular Devices) following overnight exposure to each derivative. This analysis combines the following measurements: number of primary neurites, number of branches, mean process length, and maximum process length to determine a summary of total outgrowth per cell. The only derivative identified to impact neurite outgrowth was (*S*)-LCM, which reduced neurite outgrowth by ~35% compared to vehicle (<0.01% DMSO). (*R*)-LCM was originally determined to be stereoselective, as the (*S*)-isomer requires much higher concentrations to halt seizure activity *in vivo* (Andurkar et al., 1999; LeTiran et al., 2001). This data suggests that (*S*)-LCM may retain the ability to target CRMP2.

**Table 1:** **Comparison of the ability of lacosamide derivatives to affect VGSC and CRMP2 function**.

**Compound**	**Structure**	**Slow inactivation IC_50_ (μM)^a^**	**Total outgrowth (% of vehicle)^b^**
(*R*)-LCM	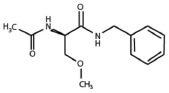	85	81.6 ± 3.7^*^
(*S*)-LCM	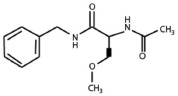	>1000	66.2 ± 5.9^*^
Derivative 1	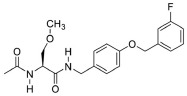	>2400	95.8 ± 4.3
Derivative 2	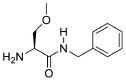	>1000	98.0 ± 4.5

a *Slow inactivation IC_50_ values were obtained from previous reports (Brittain et al., 2009; Sun et al., 2010)*.

b *Total outgrowth represents outgrowth following a 24 h incubation in 300 μM of each compound. For ease of comparison, values were normalized to vehicle*.

* *(p < 0.05 vs. vehicle) (Student's t-test) (values represent mean ± s.e.m.) (n = 263–394 cells from 8 separate culture wells)*.

### Binding of (S)-LCM to wildtype, but not mutant, CRMP2

**Figure 3:**
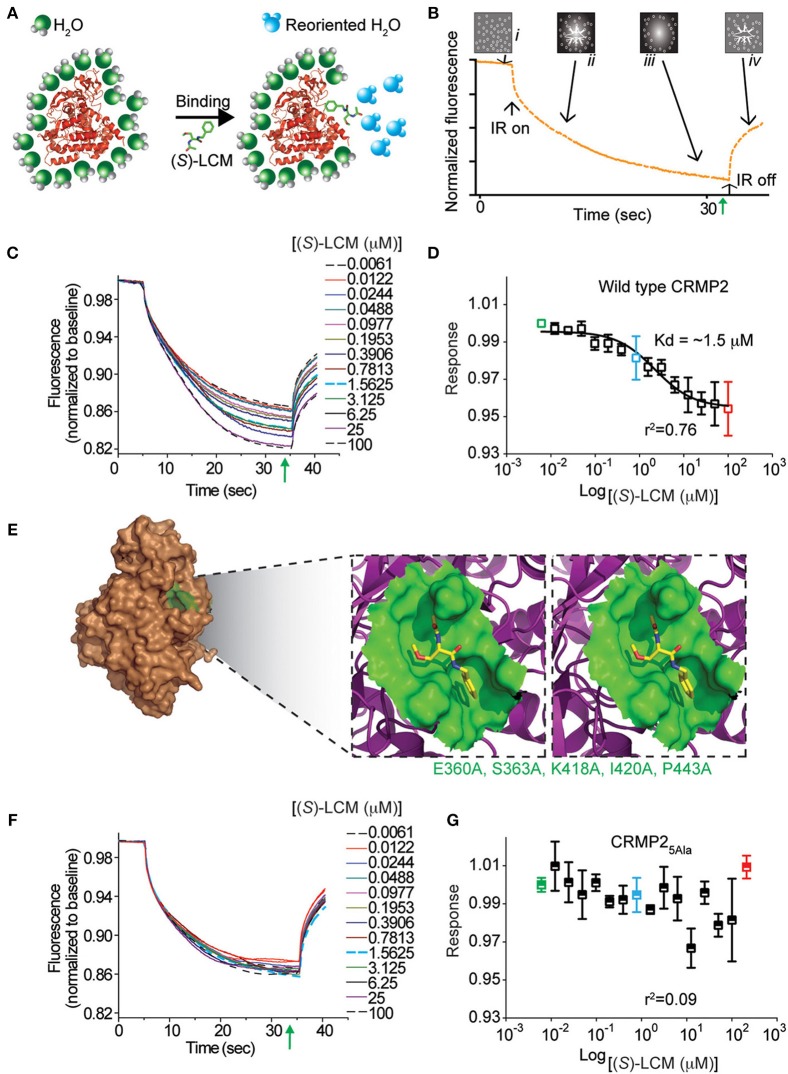
**(*S*)-LCM binds to wildtype CRMP2 in solution measured by microscale thermophoresis (MST). (A)** Illustration of hydration shell changes after small molecule [i.e., (*S*)-LCM] binding to a macromolecule. The changes in the hydration shell properties are detected in the MST instrument. **(B)** MST assay principle. The four stages in a thermophoresis experiment: (i) initial state (all molecules are randomly distributed); (ii) infrared (IR) laser is turned on, and thermophoresis commences; (iii) steady state flow while IR laser is turned on; and (iv) equilibration to initial state by back-diffusion with IR laser turned off. The green arrow represents the steady-state time point at which the MST measurements were analyzed for the graphs shown in **(D,G)**. **(C)** MST time traces of concentrations of (*S*)-LCM ranging from 0.006 to 100 μM. Increasing concentrations altered thermodiffusion of NT-647 labeled CRMP2. **(D)** MST values were used to determine dissociation constant for binding of (*S*)-LCM to wildtype CRMP2, apparent *K*d = 1.5 ± 0.01 μM; the curve was fit with an *r*^2^ value of 0.76. **(E)** Stereo view of the binding site for *(S*)-LCM within one monomer of the CRMP2 structure (Stenmark et al., 2007; Wang et al., 2010b). (*S*)-LCM is shown in capped-sticks representation. The amino acids mutated to alanines are indicated in green text. **(F)** MST time traces of concentrations of (*S*)-LCM ranging from 0.006 to 100 μM. MST experiments were repeated using NT-647 labeled CRMP2_5ALA_ harboring mutations in residues as indicated in **(E)**. **(G)** No association could be detected between (*S*)-LCM and CRMP2_5ALA_. The data could not be fitted with a curve (*r*^2^ = 0.09). A representative of range of data points obtained from at least 3 measurements is shown.

MST was used to determine if (*S*)-LCM could interact with purified CRMP2. Using an infrared laser, precise microscopic temperature gradients are generated within thin glass capillaries filled with a fluorescently labeled protein sample (i.e., CRMP2), and the atomistic movement of molecules along these temperature gradients is monitored in the presence of increasing concentrations of an unlabeled binding partner (*S*)-LCM (Figures 3A,B). As the concentration of (*S*)-LCM increased, it bound to CRMP2 thermodiffusing out of the heated infrared spot, resulting in a change in the MST signal and providing a readout of the binding between the CRMP2 and (*S*)-LCM. NT647-labeled CRMP2 protein was incubated with varying concentrations of (*S*)-LCM (0.006–100 μM) and apparent Kd values were obtained by fitting curves using the Hill method. MST experiments revealed that (*S*)-LCM bound to WT CRMP2 with an apparent Kd of 1.5 ± 0.01 μM (Figures 3C,D). Importantly, Kd of (*S*)-LCM is similar to what is observed for (*R*)-LCM in this assay (1.0 ± 0.04 μM; Figure 3D) (Wilson and Khanna, 2014). We had previously identified 5 key residues on CRMP2, essential for coordinating (*R*)-LCM binding and mutated them to alanines to create CRMP2_5ALA_, whose function mimics that of wildtype CRMP2, yet is not impaired by the presence of *(R)*-LCM (Wang et al., 2010b; Wilson et al., 2012a) (Figure 3E). MST experiments revealed that (*S*)-LCM did not interact with NT647-labeled CRMP2_5ALA_ (Figures 3F,G), suggesting that the same binding pocket is necessary for coordinating both (*R*)- and (*S*)-LCM binding.

### (S)-LCM impairs the ability of CRMP2 to enhance tubulin polymerization without altering tubulin binding or VGSC slow-inactivation

**Figure 4:**
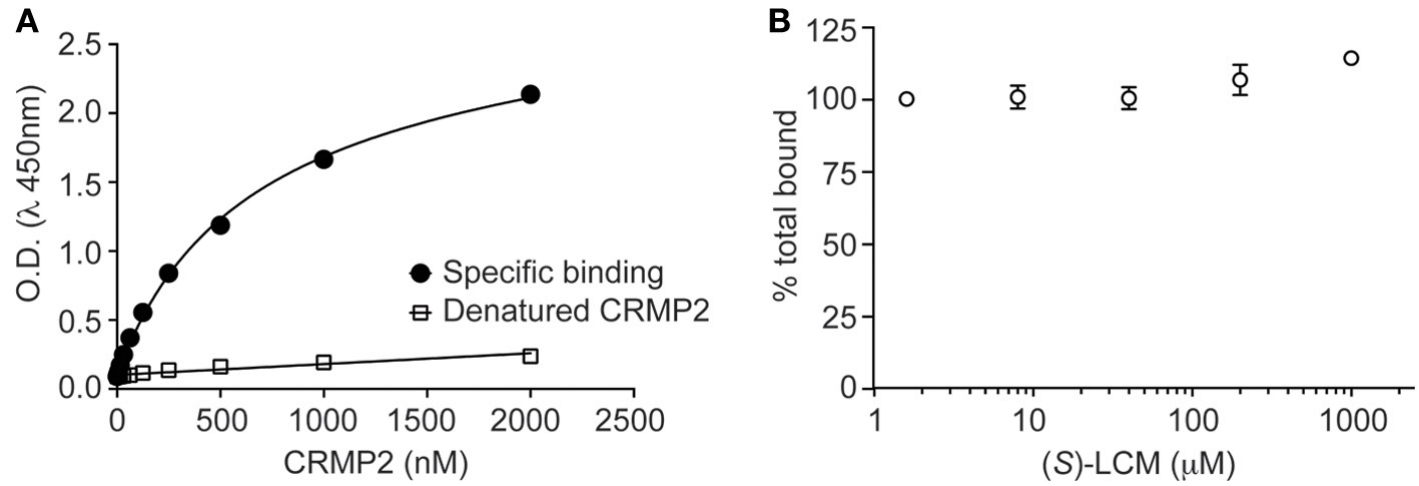
**(*S*)-LCM does not affect tubulin-CRMP2 binding. (A)** 96-well plates coated with 200 ng tubulin were incubated with increasing concentrations of recombinant CRMP2 or heat-denatured CRMP2. The *Y axis* displays the OD_450_ absorbance of the ELISA using CRMP2-specific antibodies. CRMP2 bound to tubulin with half-saturation concentration of ~607 nM. Binding of heat-denatured CRMP2 demonstrated non-saturable background binding to tubulin. **(B)** Competitive binding assay revealed that (*S*)-LCM does not abrogate the binding of CRMP2 to tubulin. For **(A,B)**, all measurements were performed in sextuplicate and error bars indicate standard error of the mean. Most of the error bars are smaller than the symbols.

It was previously determined that inhibition of CRMP2-mediated neurite outgrowth by (*R*)-LCM was mediated, not through attenuation of tubulin binding, but by impairing the ability of CRMP2 to enhance tubulin polymerization (Wilson et al., 2012a). To ensure that (*S*)-LCM functions in a similar manner, an ELISA-based competition assay was used to determine the ability of CRMP2 to bind tubulin in the presence of increasing concentrations of (*S*)-LCM. Similar to what was previously observed for (*R*)-LCM, binding of CRMP2 and tubulin was not impacted by as much as 1 mM (*S*)-LCM (Figure 4).

**Figure 5:**
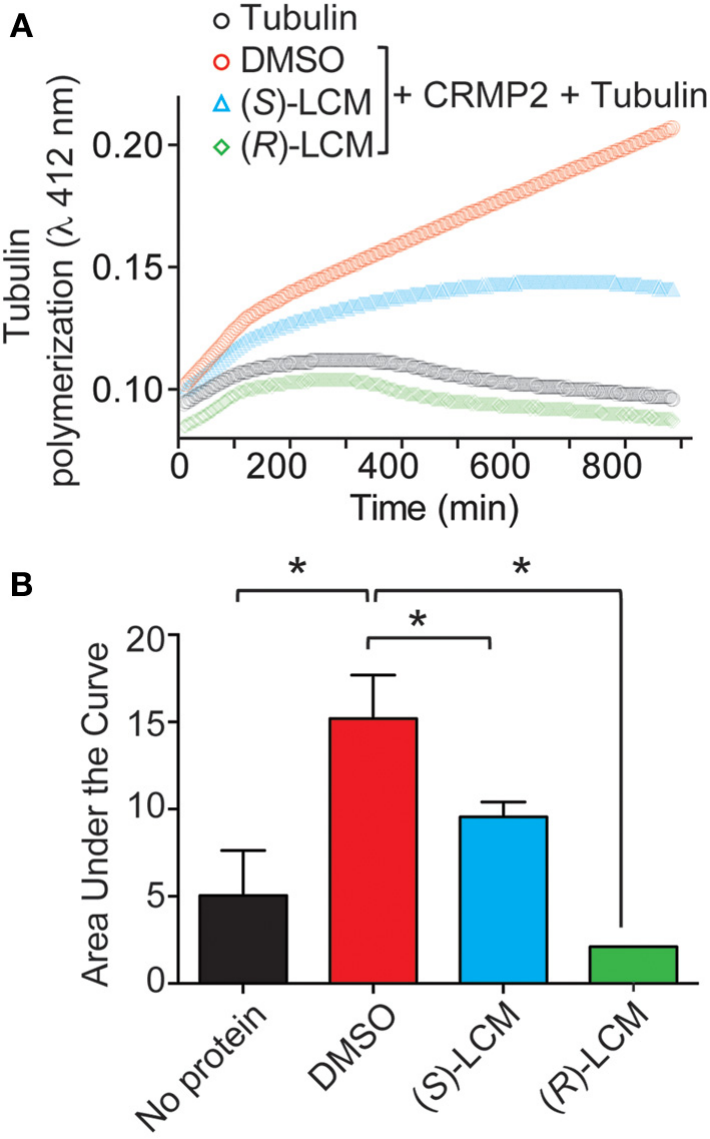
**(*S*)-LCM subverts CRMP2-mediated tubulin polymerization. (A)** Effects of enantiomers of LCM on CRMP2-mediated microtubule assembly were measured by light scattering and absorbance at 412 nm. Also shown is the basal tubulin self-polymerization in the absence of any additional protein (no protein). Values represent measurements performed in triplicate. Background absorbance was subtracted from each measurement. Values from a representative experiment are illustrated. **(B)** Average area under the curve values ± s.e.m. calculated from the tubulin polymerization curves shown in **(A)**. The addition of CRMP2 increased polymerization, while the inclusion of 3 μM (*R*)- or (*S*)-LCM led to a significant reduction in CRMP2-mediated enhancement of tubulin polymerization AUC compared to DMSO (^*^p < 0.05; One-Way ANOVA, Bonferroni *post-hoc* analysis).

Subsequently, the ability of CRMP2 to enhance tubulin polymerization in the presence of (*S*)-LCM was examined via turbidimetric assay. Based on the principle that light is scattered by microtubules to an extent that is proportional to the concentration of microtubule polymer, this assay determines the extent of tubulin polymerization by measuring changes in absorbance. Consistent with previous results (Wilson et al., 2012a), the addition of 200 nM recombinant CRMP2 protein dramatically enhanced tubulin polymerization by ~2.8-fold as demonstrated by area under the curve measurements compared to tubulin alone (*p* < 0.05) (Figures 5A,B). Similar to what is observed for (*R)-LCM*, the ability of CRMP2 to enhance tubulin polymerization was impaired by ~44.0% by as little as 3 μM (*S*)-LCM (*p* < 0.05) (Figure 5B). Additionally, FM4-64 labeling revealed that (*S*)-LCM does not impact synaptic bouton size—a phenomenon regulated by CRMP2 (Supplemental Figure 2).

**Figure 6:**
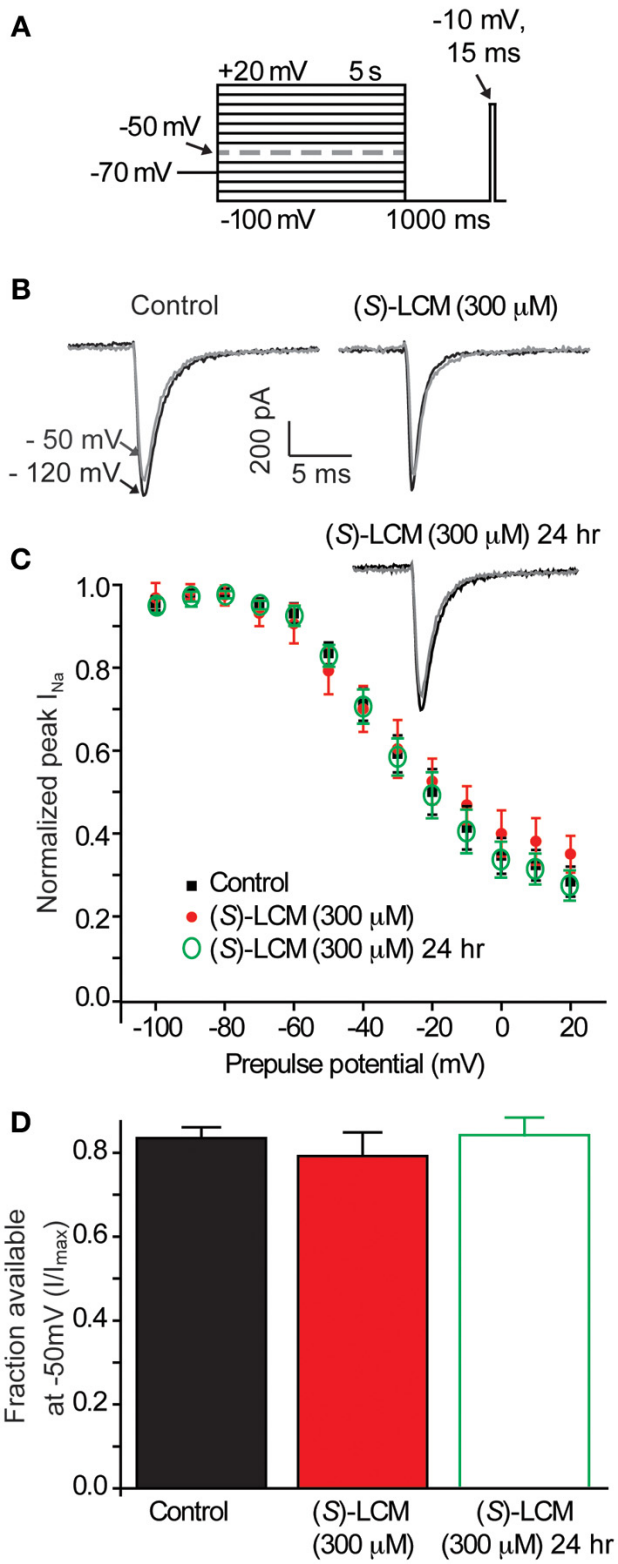
**(*S*)-LCM does not alter slow-inactivation of voltage-gated sodium channels. (A)** Voltage protocol for slow inactivation. Currents were evoked by 5 s prepulses between −100 and +20 mV and then fast-inactivated channels were allowed to recover for 5 s at a hyperpolarized pulse to −100 mV. The fraction of channels available was determined by a 15 ms test pulse at −10 mV. **(B)** Representative peak Na^+^ currents, in response to a step to −10 mV following a prepulse at −100 mV (black trace) and −50 mV (gray trace) in neurons in the absence (left) or presence (right) of 300 μM (*S*)-LCM. **(C)** Summary of steady-state slow inactivation curves from cortical neurons in the absence or presence of 300 μM (*S*)-LCM applied acutely or overnight. **(D)** For comparison, the fraction of current available following a −50 mV prepulse is depicted. (*S*)-LCM did not alter sodium channel steady-state slow inactivation in cortical neurons at either time (*p* > 0.05) (Student's *t*-test) (*n* = 6).

To ensure that (*S*)-LCM is unable to alter VGSC function at concentrations well above those required for CRMP2 binding, whole cell recordings were used to measure levels of slow inactivation (Figure 6A). Neither acute nor chronic (24 h) administration of 300 μM (*S*)-LCM altered the onset or extent of slow inactivation (Figures 6B,D). This concentration was chosen as it greatly surpasses those used for subsequent experiments.

### (S)-LCM phenocopies the effect of CRMP2 siRNA on neurite outgrowth

**Figure 7:**
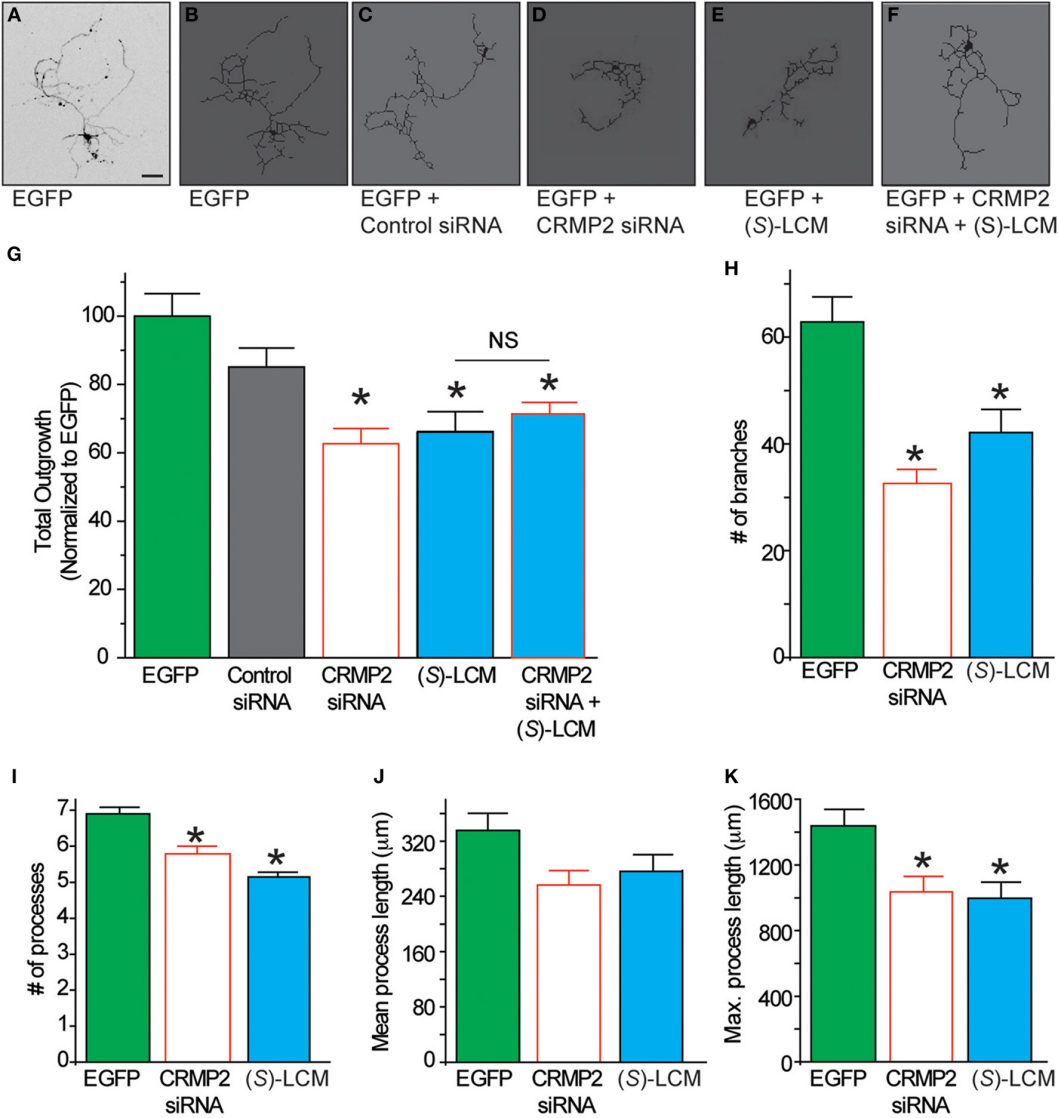
**The effect of (*S*)-LCM on neurite outgrowth phenocopies siRNA knockdown of CRMP2. (A)** Representative inverted black and white representative image of a cortical neuron 48 h following EGFP-transfection. **(B–F)** Representative tracings of neurons transfected with EGFP ± control siRNA, CRMP2 siRNA, 200 μM (*S*)-LCM, or CRMP2 siRNA + (*S*)-LCM. **(G)** Total outgrowth of neurons transfected with EGFP, control siRNA, or CRMPsiRNA combined with 24 h (*S*)-LCM treatment (200 μM). CRMP2 siRNA and (*S*)-LCM reduced outgrowth to a similar level. Combination of CRMP2 and (*S*)-LCM did not produce a further reduction. **(H–K)** Comparison of the effects of CRMP2 siRNA and (*S*)-LCM on # of branches, # of processes, mean process length, and maximum process length. (^*^p < 0.05 vs. EGFP alone, One-Way ANOVA, Tukey's *post-hoc* analysis) (values represent mean ± s.e.m.) (*n* = 86–320 cells, 8 separate culture wells) (scale bar = 50 μm).

Having demonstrated that (*S*)-LCM interacts with CRMP2, we next investigated the possible effect of this interaction on CRMP2 function. If (*S*)-LCM acts to inhibit CRMP2-mediated neurite outgrowth, then its action should mimic the phenotype bestowed by CRMP2 siRNA without the off-target effects on other CRMP2-dependent signaling pathways. Consequently, neurite outgrowth was measured from EGFP-transfected primary cultured cortical neurons using the ImageXpress Micro and MetaXpress software systems (Molecular Devices). Consistent with previous reports (Wilson et al., 2012a), siRNA knockdown of CRMP2 led to a ~37% decrease in total outgrowth (62.6 ± 4.5) compared to control (100 ± 6.6) (see Figures 7A–G). Importantly, neurite outgrowth was not altered by control siRNA (85.1 ± 5.6) (*p* > 0.05). CRMP2 siRNA-mediated reduction in neurite outgrowth was recapitulated by overnight application of 200 μM *(S)*-LCM, which decreased total outgrowth by ~34% compared to control (66.2 ± 4.5) (*p* < 0.05) (Figures 7A–K). The effects of (*S*)-LCM and CRMP2 siRNA appeared to mutually occlude one another as *(S)*-LCM was not able to provide a further reduction following CRMP2 knockdown [(71.3 ± 3.3) vs. (62.6 ± 4.5)] (*p* > 0.05) (see Figures 7A–G). Total outgrowth is a composite summary of the following parameters: number of branches, number of processes, mean process *length*, and maximum process length (Figures 7H–K), all of which, aside from mean process length, were reduced by both *(S)*-LCM and CRMP2 siRNA, compared to both control siRNA and no treatment. Combined treatment of CRMP2 siRNA and (*S*)-LCM did not produce a further reduction in any parameter (data not shown).

### Targeting CRMP2 prevents activity dependent neurite outgrowth

**Figure 8:**
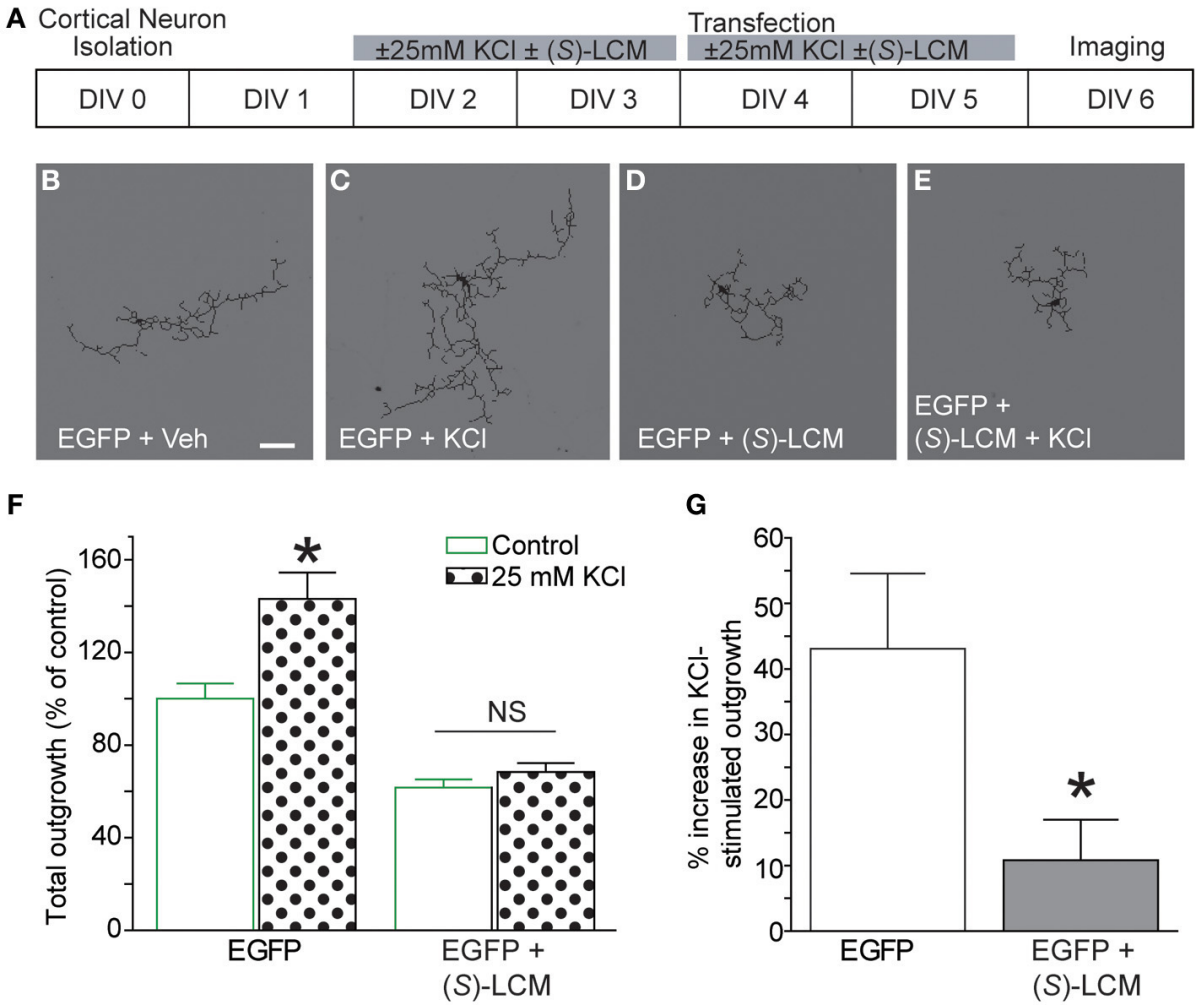
**Targeting CRMP2 prevents activity-dependent increase in neurite outgrowth. (A)** Timeline of experimental procedures. **(B–E)** Representative tracings of cortical neurons expressing EGFP and incubated for 96 h in vehicle, 25 mM KCl, 200 μM (*S*)-LCM, or 25 mM KCl + 200 μM (*S*)-LCM. **(F)** Total outgrowth of cortical neurons exposed to 25 mM KCl in the presence or absence of 200 μM (*S*)-LCM. **(G)** In naïve neurons, KCl exposure increased outgrowth by ~40% compared to vehicle. Co-application of (*S*)-LCM blunted the KCl-induced increase to ~10% (^*^p < 0.05 compared to control; Student's *t*-test) (*n* = 110–379 cells, across 8 separate culture wells) (scale bar = 50 μm).

Previously, CRMP2 was identified to be involved in activity-dependent neurite outgrowth of cerebellar granule cells (Tan et al., 2013). Unlike other central neurons, cerebellar granule cells require slightly depolarizing conditions for survival *in vitro*. Therefore, it is difficult to generalize this finding to other neuron populations within the central nervous system. As such, it is not known if CRMP2 is involved in outgrowth induced by depolarization in neurons where it is not necessary for survival. To determine the involvement of CRMP2 in activity-driven neurite outgrowth, cortical neurons overexpressing EGFP were exposed to 25 mM KCl and maintained for 96 h to ascertain the extent of activity dependent neurite outgrowth (Figure 8A). Consistent with previous findings (Tan et al., 2013), chronic depolarization with 25 mM KCl led to a ~43% increase in total neurite outgrowth (143.1 ± 11.5) compared to control (100 ± 6.6) (Figure 8B–G). Notably, blockade of CRMP2-mediated neurite outgrowth by (*S*)-LCM was sufficient to prevent activity dependent growth induced by KCl (68.4 ± 3.8 vs. 61.7 ± 3.5) (*p* > 0.05). As our earlier data demonstrated that (*S*)-LCM is not affecting VGSC function in these neurons, these data suggest that KCl-facilitated neurite outgrowth is dependent on CRMP2.

**Figure 9:**
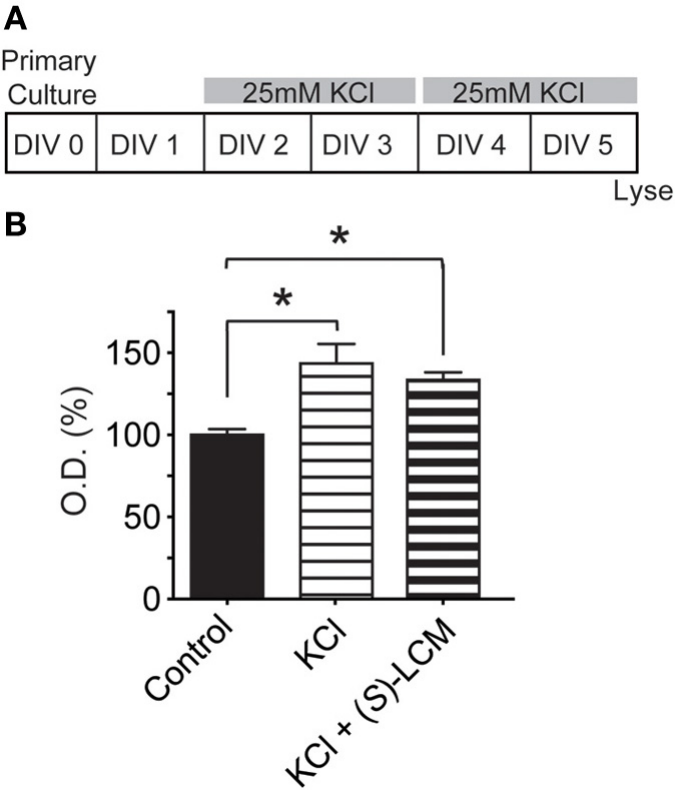
**Chronic KCl depolarization increases the association of CRMP2 and tubulin. (A)** Timeline of experimental procedures. **(B)** Summary of CRMP2-binding to tubulin from cortical cell lysates as determined by ELISA. The values obtained from these experiments are arbitrary optical densities. Thus, we have normalized the values of treated groups to the control group, which was set to 100%. This allows us to compare values across experiments. KCl exposure increased CRMP2 binding by ~43.5% compared to control. As (*S*)-LCM was previously shown not to impact CRMP2/tubulin binding, its co-application did not alter the effect of KCl. (^*^p < 0.05 compared to control; One-Way ANOVA, Tukey's *post-hoc* analysis) (*n* = 4).

To determine the potential mechanism underlying CRMP2's role in KCl-facilitated neurite outgrowth, we examined the ability of CRMP2 to bind tubulin following chronic exposure to KCl (Figure 9A). Lysates from cortical neurons that had been exposed to KCl for 96 h were incubated on tubulin-coated plates. An ELISA was then used to determine the amount of bound CRMP2. Consistent with the notion that a loss of phosphorylation should translate into an increase in CRMP2 activity, KCl exposure led to a ~43.5% increase in the binding of CRMP2 to tubulin compared to control (*p* < 0.05) (Figure 9B). As (*S*)-LCM was previously demonstrated to alter CRMP2 function by inhibiting its ability to enhance the intrinsic GTPase activity of tubulin, the addition of (*S*)-LCM did not alter the effect of KCl on CRMP2/tubulin binding [(143.5 ± 11.9) compared to KCl alone (133.5 ± 4.6)] (*p* > 0.05) (Figure 9).

## Discussion

**Figure 10:**
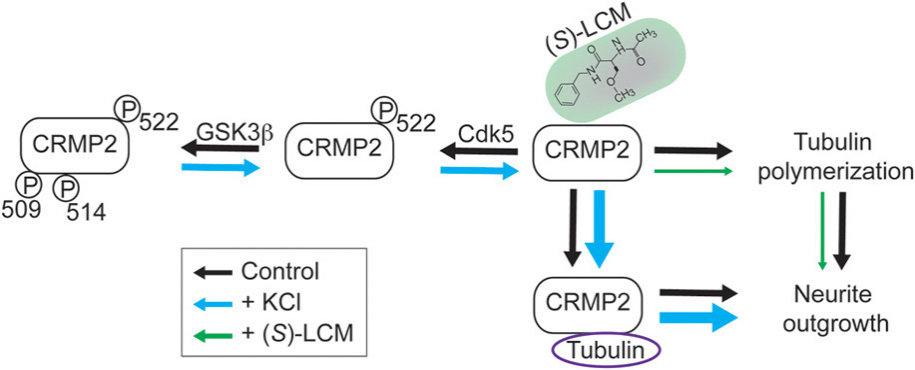
**Working model for CRMP2-mediated, activity-dependent increase in neurite outgrowth and preferential inhibition by (*S*)-LCM**. In control neurons (black arrows), CRMP2-mediated neurite outgrowth is driven by the binding of CRMP2 and tubulin, as well as, the enhancement of tubulin polymerization. Phosphorylation of CRMP2 by GSK3β and/or Cdk5 impairs its binding to tubulin, leading to neurite outgrowth arrest. Neuronal activity driven by KCl depolarization (blue arrows) leads to reduced priming by Cdk5 (residue S522), which prevents subsequent phosphorylation by GSK3β (residues T509/T514/S518; latter residue not shown). This transition to the unphosphorylated form results in increased binding of CRMP2/tubulin and CRMP2-dependent tubulin polymerization, thereby increasing neurite outgrowth. (*S*)-Lacosamide (green arrows) interacts with CRMP2 to directly impair its GAP activity, thus preventing the enhancement of tubulin polymerization and, therefore, neurite outgrowth (arrow size denotes the effect of treatment, i.e., increases in size imply a positive effect and decreases in size imply a negative effect).

Our findings demonstrate that KCl-facilitated neurite outgrowth in cultured cortical neurons is a CRMP2-dependent process. Specifically, neuronal activity led to changes in CRMP2 activity through regulation of its phosphorylation state (Figure 10). Interestingly, decreased levels of GSK3β-phosphorylated CRMP2 were observed following activity that were secondary, not to changes in GSK3β expression or activity, but rather decreased CRMP2 priming by Cdk5. CRMP2 has been suggested to be involved in many processes with potential activity-dependent components such as epilepsy, pain, and schizophrenia (Johnston-Wilson et al., 2000; Nakata et al., 2003; Czech et al., 2004; Fallin et al., 2005; Ryu et al., 2008; Brittain et al., 2011a; Wilson et al., 2012a,b). These findings may provide key insight into the importance of CRMP2 within these neuropathologies. Previous work in our lab suggested the potential anti-epileptogenic benefit of targeting CRMP2-mediated neurite outgrowth following CNS insult (Wilson et al., 2012a). The findings presented here further validate CRMP2 as a potential therapeutic target in processes involving maladaptive activity-dependent neurite outgrowth.

### Neuronal activity alters the phosphorylation state of CRMP2

Previous work revealed that phosphorylation of CRMP2 by GSK3β is altered following chronic exposure to KCl in cerebellar granule cells (Tan et al., 2013). Here, we have demonstrated that chronic activity led to decreased levels of GSK3β-phosphorylated CRMP2 in primary cortical neurons as well. GSK3β phosphorylation of CRMP2 is potentially regulated by two distinct mechanisms: (1) changes in GSK3β expression or activity or (2) changes in substrate recognition. Like many substrates, prior phosphorylation of CRMP2 by Cdk5 is required for subsequent phosphorylation by GSK3β (Cole et al., 2006), providing a level of regulation independent of GSK3β activity. Our findings suggest that loss of Cdk5 priming is responsible for the activity-driven decrease in GSK3β-phosphorylated CRMP2. Interestingly, Cdk5 phosphorylation of CRMP2 displayed a time-dependent decrease following KCl exposure while phosphorylation by GSK3β did not. It is possible that a ceiling effect on GSK3β phosphorylation of CRMP2 was reached by the amount of Cdk5 phosphorylation that was decreased after only 30 min of KCl exposure. Further reduction in Cdk5 phosphorylation of CRMP2 would therefore not result in additional change in GSK3β phosphorylation.

At this point, the mechanism underlying the change in Cdk5-phosphorylated CRMP2 is unknown. As the Cdk5 site on CRMP2 (Ser522) has been shown to be resistant to dephosphorylation (Cole et al., 2008), the involvement of protein phosphatases is unlikely. Unfortunately, mechanisms by which the activity of Cdk5 is regulated following neuronal activity are not well understood. Work by Schuman and Murase suggests that neuronal activity driven by KCl depolarization leads to a decrease in Cdk5 activity that is cofactor independent (Schuman and Murase, 2003). This work has since been corroborated in a report from the Bibb group that demonstrated activity-dependent decreases in Cdk5 activity that were p35 independent (Nguyen et al., 2007). Therefore, future work will seek to identify the events linking neuronal activity to the changes in Cdk5 phosphorylation observed in this study.

The ability of CRMP2 to mediate cytoskeletal dynamics is highly dependent upon its phosphorylation state (for review see Khanna et al., 2012). In this regard, unphosphorylated CRMP2 is considered active and growth-promoting whereby upon phosphorylation, it is rendered inactive. That CRMP2 is phosphorylated by numerous kinases allows for multiple signaling pathways to converge on a single point, the balance of unphosphorylated (active) and phosphorylated (inactive) CRMP2. Changes in kinase activity that alter this balance should thereby lead to corresponding changes in CRMP2 activity. Therefore, it was not surprising that our findings revealed that the activity-dependent reduction of CRMP2 phosphorylation directly translated into an increase in binding between CRMP2 and tubulin.

### (S)-LCM is a valuable tool for targeting CRMP2-mediated neurite outgrowth in epileptogenic processes

Very few proteins can be considered truly unifunctional. The ability to carry out multiple functions allows for varying levels of dynamic regulation. A drawback of this cellular multitasking is the difficulty in dissecting out specific functions. In the case of CRMP2, the ability to regulate neurite outgrowth is merely one facet of CRMP2 function and its study can be confounded by the role of CRMP2 in other processes such as synaptic transmission (Brittain et al., 2009; Chi et al., 2009), excitotoxicity/neurodegeneration (Castegna et al., 2002; Cole et al., 2006; Sultana et al., 2007; Brittain et al., 2011b, 2012), and migration (Sun et al., 2010), among many others. Therefore, the identification of tools to parse out select functions is invaluable in the dissection of CRMP2's roles within the nervous system. Our findings indicate that (*S*)-LCM can be used in place of genetic knockdown strategies to preferentially study neurite outgrowth mediated by CRMP2. Aside from a tool for the study of this particular function of CRMP2, (*S*)-LCM may have therapeutic potential for diseases in which aberrant neurite outgrowth is known to contribute to the pathology. As more knowledge is gained regarding the role of CRMP2-mediated neurite outgrowth in various pathologies, the ability of (*S*)-LCM to abrogate this phenomenon may prove extremely valuable.

Activity-dependent neurite outgrowth holds important implications for many pathological conditions, perhaps the most apparent being epilepsy. Therefore, CRMP2 may prove a promising target for therapeutic intervention. Indeed, previous work has demonstrated that targeting CRMP2 via (*R*)-LCM prevents the aberrant increase in excitatory connectivity in an animal model of posttraumatic epileptogenesis (Wilson et al., 2012a). We also previously showed that following traumatic brain injury (TBI), CRMP2 phosphorylation is decreased concomitantly with increased mossy fiber sprouting in the hippocampus. This biological event is related to tubulin polymerization promoted by non-phosphorylated, active CRMP2. Here, we demonstrate that specific binding of (*S*)-LCM to CRMP2 results in an inhibition of CRMP2-dependent tubulin polymerization *in vitro* (Figure 5). Since (*S*)-LCM does not alter slow-inactivation of VGSCs (Figure 6), this compound has fewer off-target effects than its enantiomer parent compound (*R*)-LCM. This assertion is further supported by the observations that (*S*)-LCM is more efficient than (*R*)-LCM in inhibiting neurite outgrowth (Table 1), a CRMP2-dependent event. Thus, we propose that (*S*)-LCM can be a molecule of particular interest in targeting aberrant CRMP2-mediated neurite outgrowth in epileptogenesis. As a proof-of-concept, we previously demonstrated that (*S*)-LCM treatment, in a TBI animal model, resulted in a complete inhibition of mossy fiber sprouting compared to sham control animals (Wilson et al., 2014).

While the action of (*S*)-LCM is likely mediated by its ability to impair the enhancement of tubulin polymerization by CRMP2, the effect of (*S*)-LCM on *every* function of CRMP2 remains untested. At this time, (*S*)-LCM does not appear to alter tubulin binding nor synaptic bouton size. Additionally, the parent compound (*R*)-LCM also has no effect on these processes, as well as calcium channel trafficking or glutamate release (Wilson et al., 2012a)—processes altered by changes in CRMP2 function.

### Activity-driven neurite outgrowth in cultured cortical neurons is a CRMP2-dependent process, mediated by changes in phosphorylation state

Consistent with the observed changes in CRMP2 phosphorylation and tubulin binding, inhibition of CRMP2 by (*S*)-LCM prevented the activity-driven increase in neurite outgrowth. As previously mentioned, CRMP2 is capable of mediating neurite outgrowth through two distinct mechanisms: (1) the binding and transport of tubulin dimers (Fukata et al., 2002) and (2) enhancement of the GTPase activity and, therefore, polymerization of tubulin (Chae et al., 2009). While the impact of neuronal activity on the ability of CRMP2 to enhance tubulin polymerization is unknown, our results suggest that an increased association with tubulin accounts for the increase in CRMP2-mediated neurite outgrowth. The ability of (*S*)-LCM to preclude the effect of KCl on neurite outgrowth lies not within preventing the KCl-facilitated increase in tubulin binding, but rather via simultaneously impairing the ability of CRMP2 to promote tubulin polymerization. This data suggests that the CRMP2's inability to enhance tubulin's GTPase activity, may overrule an increase in affinity for tubulin. Nevertheless, our findings do not rule out the possibility that (*S*)-LCM's full mechanism of action may include inhibition or enhancement of CRMP2 binding with unknown partner protein(s). Our findings (Figure 10), along with those of Tan et al. (2013), represent two separate reports of the involvement of CRMP2 in activity-driven neurite outgrowth in two distinct cell populations.

## Author contributions

Participated in Research Design—Sarah M. Wilson, Aubin Moutal, Yuying Wang, Ohannes Melemedjian, May Khanna, Rajesh Khanna. Conducted Experiments—Sarah M. Wilson, Aubin Moutal, Yuying Wang, Weina Ju, Ohannes Melemedjian, Liberty François-Moutal, May Khanna, Rajesh Khanna. Performed Data Analysis—Sarah M. Wilson, Aubin Moutal, Yuying Wang, Ohannes Melemedjian, Liberty François-Moutal, Rajesh Khanna. Wrote the Manuscript—Sarah M. Wilson, Rajesh Khanna.

### Conflict of interest statement

The authors declare that the research was conducted in the absence of any commercial or financial relationships that could be construed as a potential conflict of interest.

## Abbreviations

CRMP2: collapsin response mediator protein 2
DIV: days *in vitro*
E19: embryonic day 19
ELISA: enzyme-linked immunosorbent assay
EGFP: green fluorescent protein
LCM: lacosamide
VGSC: voltage-gated sodium channel
GSK3β: glycogen synthase kinase 3 β
Cdk5: cyclin-dependent kinase 5
cAMP: cyclic adenosine monophosphate
RhoK: Rho kinase
CaMKII: calcium/calmodulin dependent protein kinase II
PP1/2A: protein phosphatase 1/2A
MST: microscale thermophoresis.
