# Supplementary material for: The functionalized amino acid (S)-Lacosamide subverts CRMP2-mediated tubulin polymerization to prevent constitutive and activity-dependent increase in neurite outgrowth
Source: Front Cell Neurosci. 2014 Jul 24;8:196. doi: 10.3389/fncel.2014.00196 (PMC4109617; doi:10.3389/fncel.2014.00196)
Supplement: Supplementary file 1 [file Presentation1.PDF]

Supplementary Material

The functionalized amino acid (*S*)-Lacosamide subverts CRMP2-mediated tubulin polymerization to prevent constitutive and activity-dependent increase in neurite outgrowth

Sarah M. Wilson[#], Aubin Moutal[†], Ohannes Melemedjian[†], Yuying Wang[¥], Weina Ju[#,‡], Liberty Francois-Moutal[†], May Khanna[†], and Rajesh Khanna[#,†]*

[#]Paul and Carole Stark Neurosciences Research Institute, Indiana University School of Medicine, Indianapolis, IN 46202, USA.
[¥]Department of Physiology and Pathophysiology, College of Medicine, Xi'an Jiaotong University, Shaanxi, Peoples Republic of China.
[‡]Department of Neurology, The First Hospital of Jilin University, and Jilin University, 71 Xin Min Street, Peoples Republic of China.
[†]Department of Pharmacology, College of Medicine, University of Arizona, Tucson, AZ 85742, USA.

*Correspondence: Dr. Rajesh Khanna
Department of Pharmacology, College of Medicine,
University of Arizona
1501 North Campbell Drive,
P.O. Box 245050,
Tucson, AZ 85724, USA,
Office phone: (520) 626-4281; Fax: (520) 626-2204; Email: rkhanna@email.arizona.edu

1. Supplementary Methods. *FM4-64 imaging.* This was completed exactly as described (1). FM dye imaging was performed on cortical cultures 7 days after plating. Presynaptic terminals were loaded with the fixable analog of the fluorescent styryl dye N-(3-triethylammoniumpropyl)-4-(6-(4-(diethylamino)phenyl)hexatrienyl)pyridinium dibromide (FM4-64; 15 μM) by incubation of the cells for 1 min in high K^+ solution containing 58 mM NaCl, 90 mM KCl, 10 mM HEPES, 3 mM $CaCl_2 \cdot 2H_2O$, 8 mM glucose, and 2 mM $MgCl_2 \cdot 6H_2O$ (pH 7.3). The cells were then washed in Ca^{2+}-free solution for 2 min to reduce nonspecific staining. The nerve terminals were identified under a confocal microscope (Nikon Livescan SFC inverted microscope) using an oil immersion CFI Plan APO VC x60 objective lens. Fluorescence of FM dye was excited at 543 nm. Quantitative measurements of FM4-64 fluorescence intensity at individual synapses were obtained by averaging a 4 × 4 square area of pixel intensities centered on the optical center of a given fluorescent puncta. Individual puncta was selected by hand, and the optical center of mass used to center the measurement box was calculated over a slightly larger area (typically 10 × 10 pixels). Large puncta, typically representative of clusters of smaller synapses, were not analyzed during the selection procedure as were any puncta that were not clearly discernible during the time course of the experiment. Areas for imaging contained networks of well-defined dendrites and no cell bodies. At least 100 boutons from each condition were imaged in each of these experiments.

2. **Supplementary Figures.**

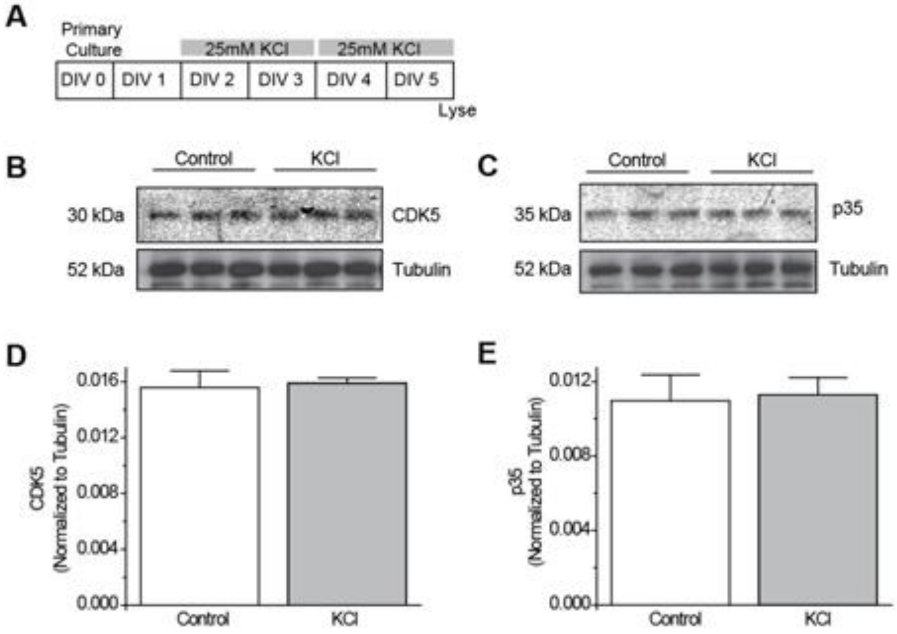

Supplementary Figure 1. KCl-induced activity does not alter expression of CDK5 or p35. (A) Timeline of experimental procedures. (B-C) Western blots of p35 and CDK5 levels from naïve cortical neurons compared to those exposed to KCl for 96 hr. (D-E) Levels of p35 and CDK5 expression were not altered following KCl treatment. (Student's *t*-test) (values represent mean ± SEM) (n = 4-5).

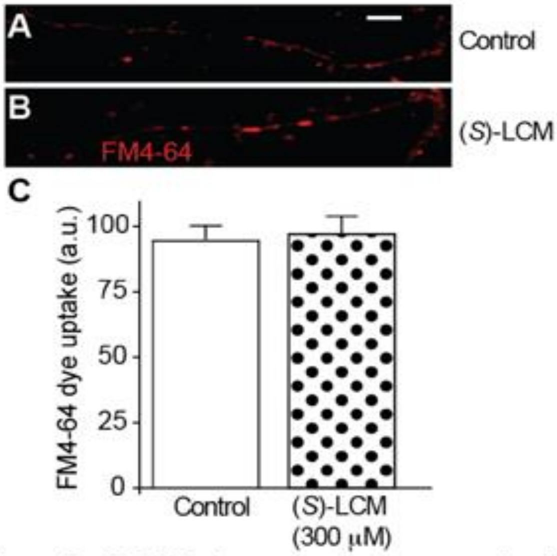

<u>Supplementary Figure 2.</u> **(*S*)-LCM does not affect synapse density in cortical neurons.** Representative images of synapses from untreated (**A**) or (**B**) (*S*)-LCM treated (200 μM, 3 days) cortical neuron neurites following a 1 min loading with the fixable styryl dye FM4-64 (15 μM) in excitatory Tyrode's solution. (**C**) Average FM4-64 dye uptake, in arbitrary units (a. u.), is plotted as mean ± SEM. Synapses exhibited a similar level of loading in either condition ($p > 0.05$; Student's t-test). A total of 112 (n= 6 neurons for control) and 127 puncta (n=7 for (S)-LCM-treated neurons) were counted.

3. References[1]

1. Brittain JM, Piekarz AD, Wang Y, Kondo T, Cummins TR, Khanna R. An atypical role for collapsin response mediator protein 2 (CRMP-2) in neurotransmitter release via interaction with presynaptic voltage-gated calcium channels. *The Journal of biological chemistry* (2009) **284**(45):31375-90. Epub 2009/09/17. doi: 10.1074/jbc.M109.009951. PubMed PMID: 19755421; PubMed Central PMCID: PMC2781534.
